# Robustness of pulmonary nodule radiomic features on computed tomography as a function of varying radiation dose levels—a multi-dose *in vivo* patient study

**DOI:** 10.1007/s00330-023-09643-8

**Published:** 2023-04-19

**Authors:** Gijs A. Bartholomeus, Wouter A. C. van Amsterdam, Annemarie M.den Harder, Martin J. Willemink, Robbert W. van Hamersvelt, Pim A. de Jong, Tim Leiner

**Affiliations:** 1 University Medical Center Utrecht, Utrecht, the Netherlands; 2 Department of Radiology, Stanford University School of Medicine, Stanford, CA USA; 3 Mayo Clinic, Rochester, MN USA

**Keywords:** Humans, Linear models, Tomography (x-ray computed), Multiple pulmonary nodules, Radiation dosage

## Abstract

**Objective:**

Analysis of textural features of pulmonary nodules in chest CT, also known as radiomics, has several potential clinical applications, such as diagnosis, prognostication, and treatment response monitoring. For clinical use, it is essential that these features provide robust measurements. Studies with phantoms and simulated lower dose levels have demonstrated that radiomic features can vary with different radiation dose levels. This study presents an in vivo stability analysis of radiomic features for pulmonary nodules against varying radiation dose levels.

**Methods:**

Nineteen patients with a total of thirty-five pulmonary nodules underwent four chest CT scans at different radiation dose levels (60, 33, 24, and 15 mAs) in a single session. The nodules were manually delineated. To assess the robustness of features, we calculated the intra-class correlation coefficient (ICC). To visualize the effect of milliampere-second variation on groups of features, a linear model was fitted to each feature. We calculated bias and calculated the *R*^2^ value as a measure of goodness of fit.

**Results:**

A small minority of 15/100 (15%) radiomic features were considered stable (ICC > 0.9). Bias increased and *R*^2 ^decreased at lower dose, but shape features seemed to be more robust to milliampere-second variations than other feature classes.

**Conclusion:**

A large majority of pulmonary nodule radiomic features were not inherently robust to radiation dose level variations. For a subset of features, it was possible to correct this variability by a simple linear model. However, the correction became increasingly less accurate at lower radiation dose levels.

**Clinical relevance statement:**

Radiomic features provide a quantitative description of a tumor based on medical imaging such as computed tomography (CT). These features are potentially useful in several clinical tasks such as diagnosis, prognosis prediction, treatment effect monitoring, and treatment effect estimation.

**Key Points:**

*• The vast majority of commonly used radiomic features are strongly influenced by variations in radiation dose level.*

*• A small minority of radiomic features, notably the shape feature class, are robust against dose-level variations according to ICC calculations.*

*• A large subset of radiomic features can be corrected by a linear model taking into account only the radiation dose level.*

**Supplementary Information:**

The online version contains supplementary material available at 10.1007/s00330-023-09643-8.

## Introduction

Advances in data science have led to a surge in imaging biomarkers to improve lung cancer diagnosis, prognostication, and treatment response monitoring. Among these modern biomarkers is the class of computed tomography (CT) radiomic features. Radiomics is defined as the quantification of CT radiographic phenotype using data-characterization algorithms [1, 2]. Statistical models are used to relate these radiomic features to diagnosis, prognostication, and treatment response.

Early detection of possibly malignant pulmonary nodules would make it possible to start therapy in an earlier stage which is prognostically favorable [3]. Conversely, early discrimination of benign nodules from malignant nodules would relieve patients from unnecessary follow-up CT scans. Thus, the goal of radiomics is to go beyond morphological imaging and to aid in the diagnosis, classification, and therapeutic decision-making of patients who undergo radiographic imaging using statistical models.

For radiomic features to be useful in the clinical process, feature values need to be reproducible. This is to say, a feature should be influenced primarily by biological traits of the patient, and not by external conditions such as the type of CT equipment, reconstruction algorithm, region of interest (ROI) selection and segmentation, etc. A drawback of radiomic features is that they seem to be sensitive to conditions currently not standardized in clinical care. One of these variables in CT scanning is the tube current–time product (milliampere-seconds, or mAs). Computed tomography is a major source of radiation exposure related to medical imaging. To reduce the dose, the level of milliampere-seconds is lowered at the cost of increased image noise [4]. Because image noise increases non-linearly with decreasing milliampere-seconds [5], we hypothesize that this increase in noise will influence radiomic feature values. Although some phantom studies have shown that the effect of varying tube current on radiomic features does not significantly affect radiomic features [6], other studies have shown milliampere-second variation does in fact significantly influence radiomic feature values [7, 8]. Although several *in vivo* dose modulation radiomic feature robustness studies have been performed to date, these studies are retrospective in the sense that they compare features taken from a single diagnostic scan, and later follow-up scans [9, 10]. As mentioned in the systematic review by Reiazi et al: “The drawbacks of the retrospective studies are that the investigators did not have control over the parameters studied, and the range of the scan acquisition parameter variations were limited to those used in imaging patients.” [11]. Our study differs from these studies in that multiple scans with different radiation doses were obtained in a single examination within a time frame of approximately 20 min.

Therefore, we sought to investigate the *in vivo* robustness of pulmonary nodule radiomic features in patients who underwent chest CT scans at four different radiation dose levels.

## Methods

### Study population and image acquisition

In this study, patients 50 years or older with 1 or more known pulmonary nodules scheduled for a follow-up chest CT were eligible for inclusion. Detailed inclusion criteria are listed in Appendix 1. IRB approval was given under reference number NL46146.041.13 [12, 13]. Participants signed a written informed consent form prior to inclusion in the study.

A 256-slice CT system (Brilliance iCT; Philips Healthcare) was used for image acquisition. Patients were asked to hold their breath at deep inspiration during each acquisition. After scout images were obtained, image acquisition was performed using our routine non-enhanced chest CT protocol, immediately followed by 3 acquisitions at reduced radiation dose levels. Automatic current selection was only used for the reference protocol and modified to the values as described for the lower-dose acquisitions. *Z*-axis dose modulation and dynamic angular dose modulation were not used to minimize variation.

All acquisitions were performed with the same length (*Z* coverage). Images were reconstructed with a slice thickness of 1 mm and an increment of 0.7 mm. Tube current–time products of 60 (reference dose), 33 (45% reduction), 24 (60% reduction), and 15 mAs (75% reduction) were used in combination with a tube voltage of 100 kV for patients with a weight less than 80 kg and a tube voltage of 120 kV for patients with a weight greater than 80 kg. Gantry rotation time was 0.33 s with a pitch of 0.758. No contrast medium was injected. Scans were reconstructed using filtered back projection (FBP). Data will be made available for non-commercial purposes upon reasonable request to the authors.

### Segmentation

For the evaluation of the stability of radiomic features of pulmonary nodules on computed tomography, pulmonary nodules were manually segmented in the open-source image processing software platform 3D Slicer (Slicer.org). Nodules were independently identified by two experienced radiologists to make sure no pulmonary nodules were missed. For each scan, a binary (3D) label map annotating the pulmonary nodules for each radiation dose level was created by manual segmentation with the help of the semiautomatic “grow from seeds” region growing volumetric segmentation algorithm [14]. Contours were generated by one author (G.B.) and independently verified by an experienced radiologist (P.J.).

### Radiomic features

The open-source python package for the extraction of radiomic features from medical imaging Pyradiomics (version 2.2.0) was used to extract the radiomic features [15]. Statistical analysis was done in R (version 4.10.2). Seven different filters were applied to the images before feature extraction (including original image, no filter). Per filter, 86 features were extracted, divided into six different feature classes. The following feature classes were extracted: shape (only for the original image); gray-level co-occurrence matrix (GLCM); gray-level dependence matrix (GLDM); first-order, gray-level run length matrix (GLRM); and gray-level size zone matrix (GLSZM) [15]. A detailed list of extracted features can be found in Appendix 2.

### Statistical analysis

Statistical analysis was performed on a nodule level, using the package psych (version 1.9.11) in R. The intra-class correlation coefficient 3.1 (ICC) was calculated to assess feature robustness [16] by assessing agreement in radiomic feature values between CT scans acquired with different radiation doses, and is calculated as follows:

$$\mathrm{ICC}=\frac{{\mathrm{MS}}_{\mathrm{R}}-{\mathrm{MS}}_{\mathrm{E}}}{{\mathrm{MS}}_{\mathrm{R}}+\left(k-1\right){\mathrm{MS}}_{\mathrm{E}}}$$

where MS_R_ = mean square for rows, MS_E_ = mean square error, and *k* = number of different radiation dose levels. According to Koo et al, ICC values less than 0.5 were considered as having poor reproducibility, values less than 0.75 as having moderate reproducibility, values between 0.75 and 0.9 as having good reproducibility, and values over 0.9 as having excellent reproducibility [17].

While the ICC metric is “ground truth agnostic,” treating every radiation dose level as being equivalent, it is arguably not the most optimal metric here. Due to the physical properties of computed tomography, a lower dose invariably leads to a worse signal-to-noise ratio. It is therefore likely that features extracted from lower-dose images contain the same or less information about the underlying biology of the nodule. We therefore performed an additional analysis where we treated the full-dose scan as a ground-truth observation. Features were scaled by the subtraction of the mean and the division by the standard deviation of the highest radiation dose (60 mAs) scans. To investigate how well ground-truth radiomic feature values can be obtained from lower-dose acquisitions using linear transformations, separate linear regression models were fitted for each feature and each reduced dose level. Feature values for 60 mAs were used as ground truth. These linear models were used to evaluate two metrics: *bias* and *R*^2^. Bias indicates the average deviation of feature values in a lower-dose setting from the average value in the full-dose (60 mAs) setting and is equal to the intercept term in a linear regression model. For each feature and for each dose level, the *R*^2^ measures how much of the variation in ground-truth values can be explained using a linear correction of the lower-dose values. An *R*^2^ value of 1 indicates that the values from the full-dose scan can be perfectly reconstructed from the lower-dose image using a linear model. A value of 0 indicates that it is impossible to reconstruct the ground-truth values from the lower-dose values using a linear model [18].

## Results

### Study population and radiomic feature extraction

**Fig. 1:**
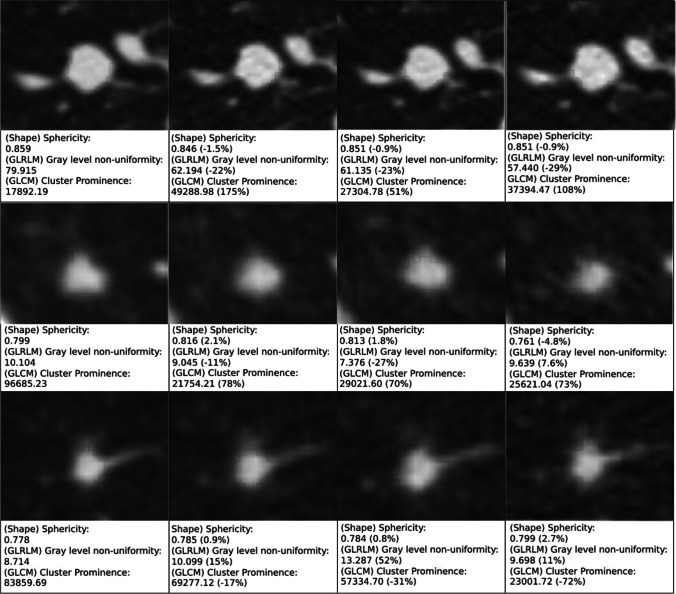
Segmentation of three nodules from (left to right) 60-, 33-, 24-, and 15-mAs scans. Below each segmentation are listed high ICC shape (0.807), high ICC non-shape (0.966), and low ICC (0.207) feature values and increase in % compared to the 60-mAs feature value

Nineteen patients were included in the study, with ages ranging from 61 to 79 years (mean age: 67 years), of which 12 were male and 7 were female. Fifteen patients had lung nodules (35 in total) of which 3 were malignant. Of the fifteen patients, three patients (2 male and 1 female, with 3 nodules) were excluded because they presented with lung masses (diameter ≥ 3 cm) instead of lung nodules [19]. In total, 12 patients with 32 nodules with a median (IQR) diameter of 7.1 (6.1–9.6) mm were included for analysis in this study. In total, 1218 features were extracted from 32*4 = 128 nodules. A graphical abstract of three nodules with exemplary feature values for the four different radiation doses is presented in Fig. 1.

### Features considered stable (ICC)

Overall, only a minority of radiomic features were reproducible. From the 100 features without a filter applied, 15 features had excellent reproducibility (ICC > 0.9), 24 features had good reproducibility (0.75 < ICC < 0.9), 31 features had moderate reproducibility (0.5 < ICC < 0.75), and 30 features had poor reproducibility (ICC < 0.5). The top 30 ICC features are listed in Table 1. ICC values for all features are listed in Appendix 3. Of note, eight out of the top ten features with highest reproducibility were shape features. Overall, ten out of fourteen shape features were found to have an ICC value greater than 0.9 and can therefore be considered stable.

**Table 1:** Top 30/100 ICCs from original filter features

**#**	Feature	Value
1	original_glrlm_GrayLevelNonUniformity	0.966
2	shape_VoxelVolume	0.96
3	shape_MeshVolume	0.96
4	original_gldm_GrayLevelNonUniformity	0.952
5	shape_SurfaceArea	0.939
6	shape_MajorAxisLength	0.935
7	shape_LeastAxisLength	0.929
8	shape_Maximum2DDiameterSlice	0.928
9	shape_Maximum2DDiameterRow	0.92
10	shape_MinorAxisLength	0.913
11	shape_Maximum2DDiameterColumn	0.907
12	shape_Maximum3DDiameter	0.904
13	original_glcm_Imc2	0.902
14	original_glszm_GrayLevelNonUniformity	0.902
15	original_glcm_Imc1	0.901
16	original_glrlm_RunLengthNonUniformity	0.885
17	original_firstorder_90Percentile	0.881
18	original_firstorder_Median	0.856
19	original_glcm_Idn	0.855
20	original_glrlm_RunLengthNonUniformityNormalized	0.854
21	original_glrlm_RunPercentage	0.845
22	original_glszm_ZonePercentage	0.845
23	original_glrlm_ShortRunEmphasis	0.843
24	shape_SurfaceVolumeRatio	0.839
25	original_gldm_DependenceEntropy	0.836
26	original_gldm_DependenceNonUniformityNormalized	0.83
27	original_firstorder_Mean	0.83
28	original_glcm_Idmn	0.813
29	shape_Sphericity	0.807
30	original_glrlm_LongRunEmphasis	0.807

### Effect of lower radiation dose on radiomicfeature values (bias – R^2^)

**Fig. 2:**
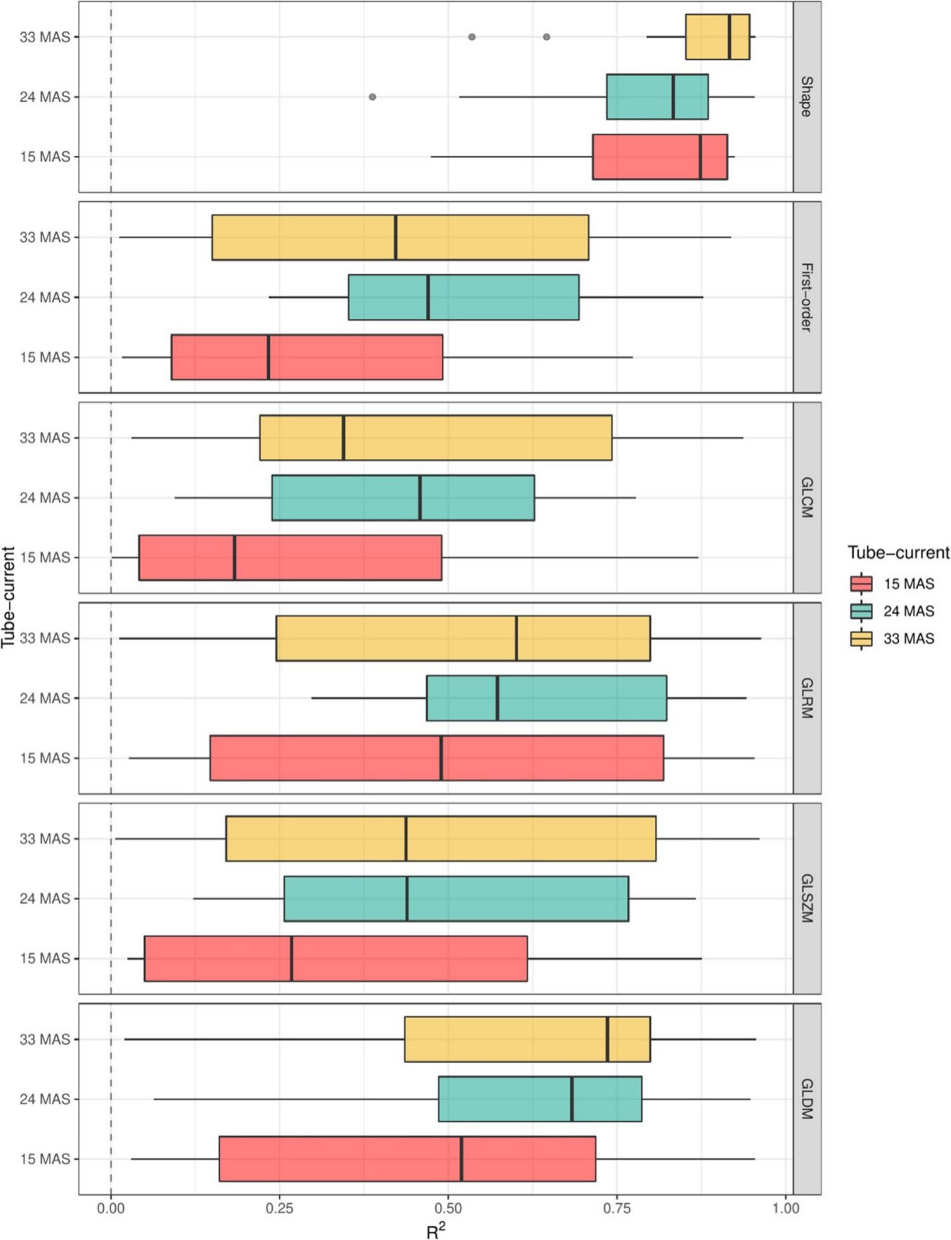
*R*^2^ boxplot filter per feature class

**Fig. 3:**
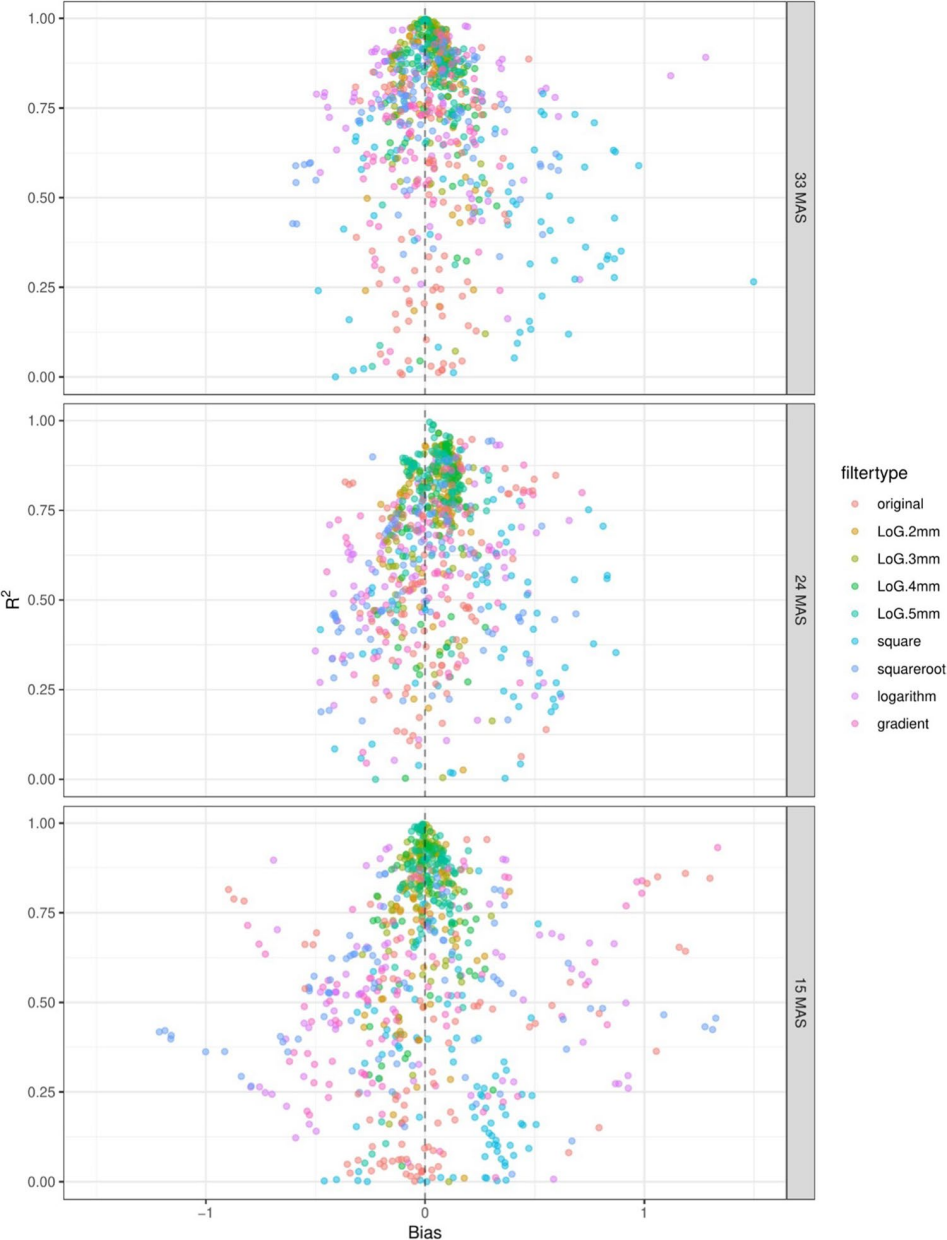
Bias vs *R*^2^ plotting for different milliampere-second levels looking at all features, colored by filters. High bias means that the value for this feature is on average higher than that for the reference dose of 60 mAs. High *R*^2^ means that the deviation of feature values can be explained very well by a linear model taking into account only the dose (mAs)

From the separate linear regression fits, bias and *R*^2^ values were extracted. These values were plotted per filter category and per feature class. In general, features showed bias which increased with decreasing dose. In addition, for most features, *R*^2^ values decreased for decreasing dose levels (Figs. 2 and 3). One percent of features had a negative slope fit. These features were omitted from the remainder of the analyses because this would imply that at a lower dose, the prognostic/diagnostic interpretation of a feature would be inverted, thus making these features unpractical in a clinical setting. None of these features was from the subset of features without a filter applied. Negative slope features are listed in Appendix 4.

Bias increased and *R*^2^ decreased with decreasing radiation dose (Figs. 2 and 3). In this analysis, the shape features were also found to have better correctability (higher *R*^2^) compared to other features.

### Robustness of features: radiomic feature classes (bias – R^2^, ICC)

**Fig. 4:**
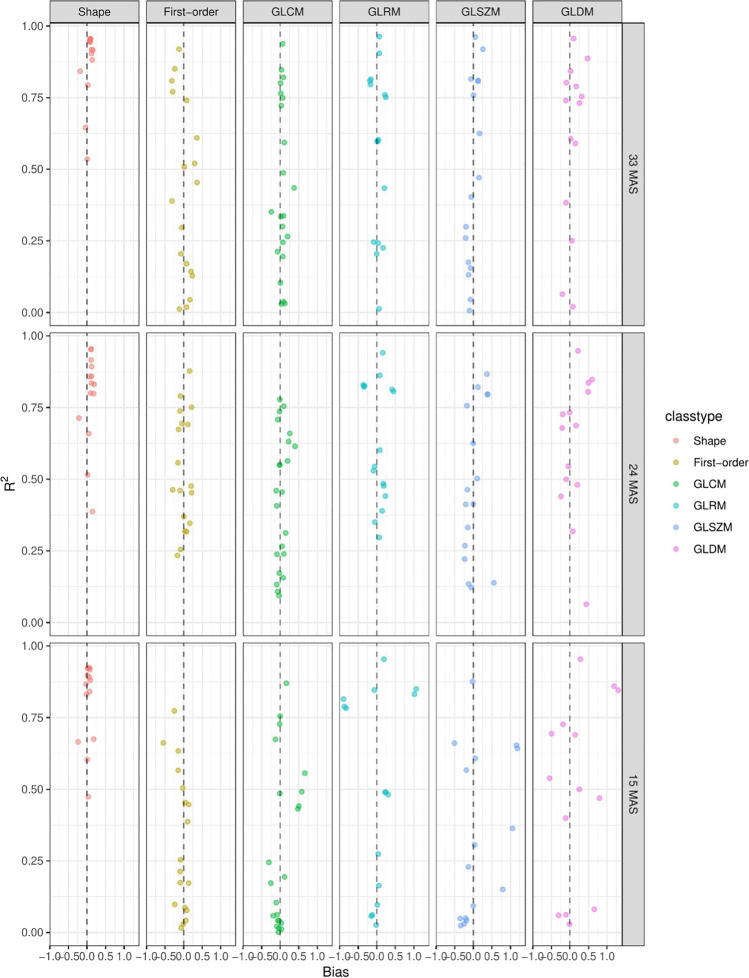
Bias vs *R*^2^ plotting for different milliampere-second levels and feature classes. High bias means that the value for this feature is on average higher than that for the reference dose of 60 mAs. *R*^2^ is a statistic that will give some information about the goodness of fit of a model. High *R*^2^ means that the deviation of feature values can be explained very well by a linear model taking into account only the dose (mAs)

To further analyze the robustness of radiomic features, the features were split in classes and bias versus *R*^2^ was plotted as a function of decreasing dose levels. The shape feature class was again found to be the most robust with the highest *R*^2^ and the lowest bias (Fig. 4). An increasing trend in bias and a decreasing trend in *R*^2^ were visible for all feature classes as a function of radiation dose. In other words, the difference from the mean of the high-dose (60 mAs) features was least for the shape feature class. Moreover, the error of shape features was fit best of all features by a linear model as a function of dose. All features were found to have an increasing difference from the mean of the high-dose features and a worse fit of the linear model, when dose level decreased.

**Fig. 5:**
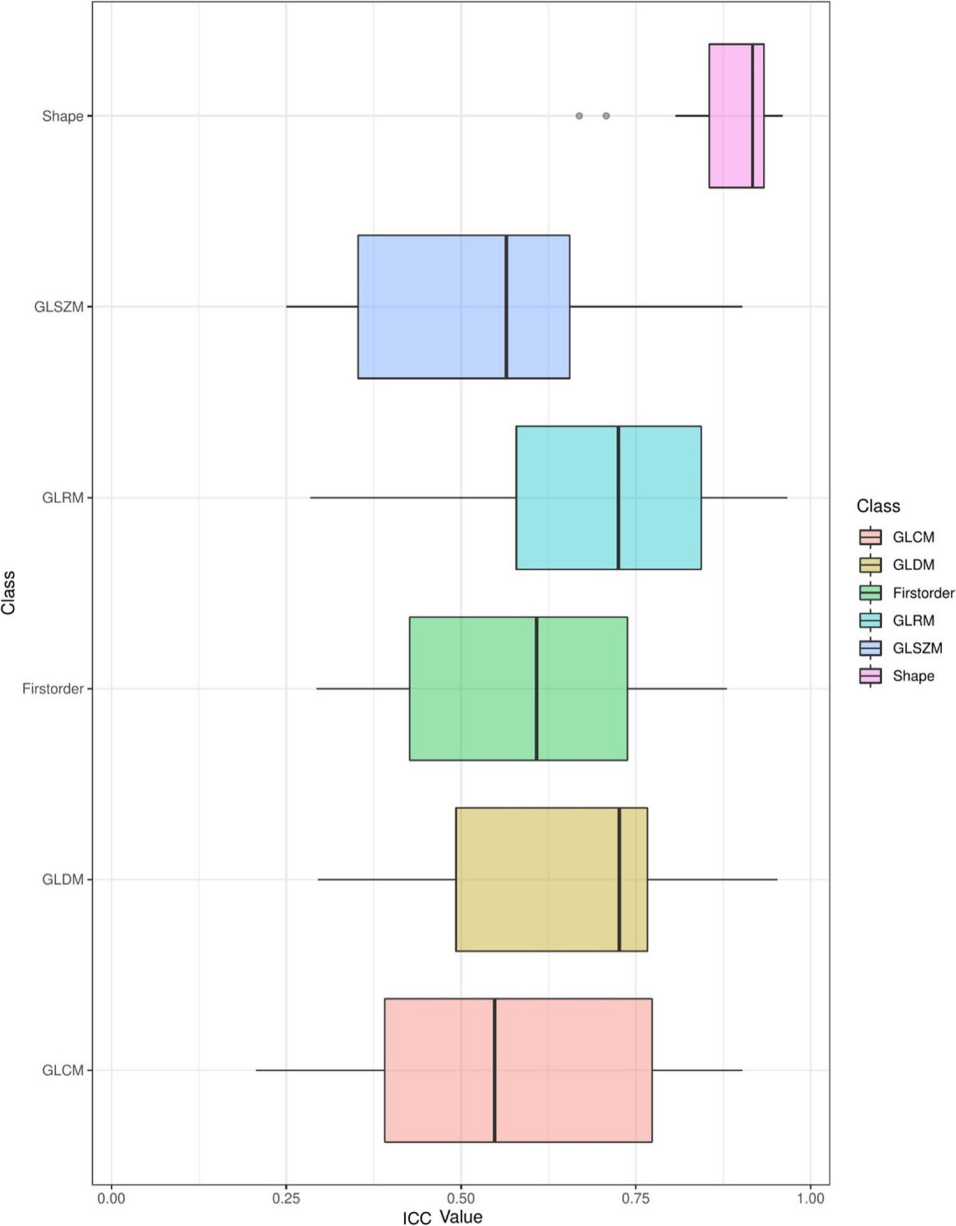
ICC boxplot; high ICC values indicate robust features

In addition, the ICC 3.1 was calculated [17]. ICC values per feature, split by feature class, are shown in Fig. 5.

Shape features had by far the highest ICC value of all feature classes, followed by GLRM features. This finding illustrates that shape features, followed by GLRM features, most strongly resemble each other in the different dose-level groups. Shape and first-order ICC, *R*^2^, and bias values are listed per feature in Tables 2 and 3.

**Table 2:** ICC, *R*^2^, and bias for shape features

Feature	ICC	*R*^2^ 15 mAs	Bias 15 mAs	*R*^2^ 24 mAs	Bias 24 mAs	*R*^2^ 33 mAs	Bias 33 mAs
VoxelVolume	0.96	0.92	0.02	0.95	0.11	0.96	0.09
Maximum3DDiameter	0.9	0.83	− 0.01	0.84	0.12	0.94	0.08
MeshVolume	0.96	0.92	0.02	0.95	0.11	0.95	0.09
MajorAxisLength	0.93	0.92	0.08	0.86	0.12	0.95	0.07
Sphericity	0.81	0.68	0.18	0.66	0.05	0.79	0.02
LeastAxisLength	0.93	0.84	0.07	0.89	0.12	0.9	0.12
Elongation	0.67	0.6	0	0.39	0.14	0.53	0
SurfaceVolumeRatio	0.84	0.67	− 0.24	0.71	− 0.22	0.84	− 0.19
Maximum2DDiameterSlice	0.93	0.92	0.06	0.83	0.19	0.92	0.16
Flatness	0.71	0.47	0.03	0.52	0.01	0.65	− 0.04
SurfaceArea	0.94	0.9	0.01	0.92	0.11	0.95	0.08
MinorAxisLength	0.91	0.89	0.06	0.8	0.18	0.92	0.12
Maximum2DDiameterColumn	0.91	0.88	0.09	0.8	0.1	0.88	0.14
Maximum2DDiameterRow	0.92	0.87	− 0.03	0.86	0.07	0.95	0.09

**Table 3:** ICC, *R*^2^, and bias for first-order features

Feature	ICC	*R*^2^ 15 mAs	Bias 15 mAs	*R*^2^ 24 mAs	Bias 24 mAs	*R*^2^ 33 mAs	Bias 33 mAs
InterquartileRange	0.43	0.48	− 0.15	0.5	0	0.93	− 0.05
Skewness	0.69	0.28	− 0.05	0.24	− 0.18	0.62	0.03
Uniformity	0.6	0.9	0.37	0.86	0.04	0.98	0.19
Median	0.86	0.51	0.45	0.82	0.23	0.62	0.1
Energy	0.7	0.67	− 0.17	0.82	0.02	0.76	0.08
RobustMeanAbsoluteDeviation	0.43	0.6	− 0.18	0.57	− 0.04	0.91	− 0.11
MeanAbsoluteDeviation	0.35	0.66	− 0.31	0.57	− 0.14	0.9	− 0.15
TotalEnergy	0.68	0.66	− 0.18	0.81	0	0.76	0.08
Maximum	0.76	0.44	− 0.41	0.34	− 0.42	0.78	− 0.18
RootMeanSquared	0.75	0.7	− 0.67	0.71	− 0.44	0.9	− 0.12
90Percentile	0.88	0.87	− 0.14	0.56	− 0.06	0.82	− 0.1
Minimum	0.32	0.66	0.86	0.69	0.51	0.91	0.22
Entropy	0.54	0.88	− 0.27	0.75	− 0.06	0.97	− 0.15
Range	0.39	0.44	− 0.55	0.36	− 0.5	0.79	− 0.22
Variance	0.29	0.53	− 0.41	0.47	− 0.22	0.88	− 0.18
10Percentile	0.61	0.67	0.75	0.7	0.44	0.88	0.16
Kurtosis	0.61	0.05	− 0.08	0.05	− 0.14	0.42	0.02
Mean	0.83	0.77	0.12	0.75	0.11	0.91	− 0.01

### Robustness of features: radiomic filters (bias – R^2^)

**Fig. 6:**
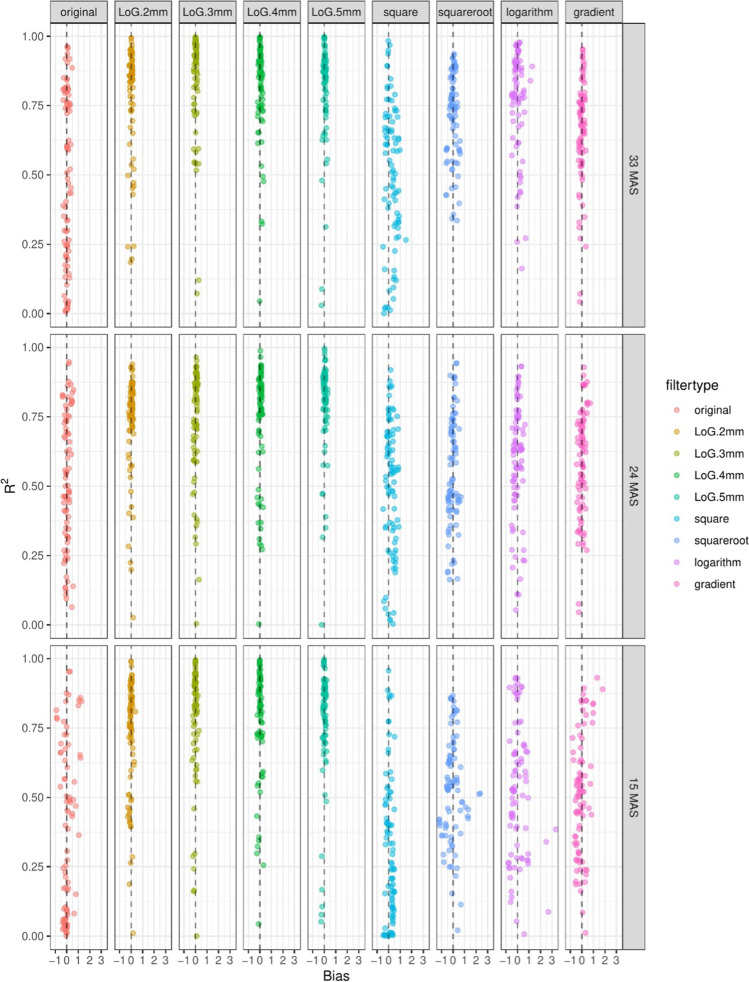
Bias vs *R*^2^ plotting for different milliampere-second levels and filters (original and wavelet). High bias means that the value for this feature is on average higher than that for the reference dose of 60 mAs. High *R*.^2^ means that the variation of feature values can be explained very well by a linear model taking into account only the dose (mAs)

Another possible variable that influences the reproducibility of radiomic features is the application of a filter to the image before feature extraction. Features calculated from filtered images were often less reproducible than those from the original image. This is demonstrated in Fig. 6, where *R*^2^ and bias plots are shown for individual features, split by image filter. Figure 6 compares the original filter to the filter classes (Laplacian of Gaussian (LoG) (sigma 2, 3, 4, 5), square, square root, logarithm, and gradient).

The trend of decrease in *R*^2^ and increase in bias were visible for all filters. Most filters were comparable to the original image regarding robustness of features. Wavelet, square, square root, logarithm, and gradient filters made the features less robust. The Laplacian of Gaussian filter seemed to make features remarkably more robust compared to the use of the original non-filtered images and other filters.

## Discussion

We performed an *in vivo*, intra-individual study on the robustness of radiomic features of pulmonary nodules as identified with computed tomography of the chest as a function of radiation dose levels. Except for shape features, we found that the majority of radiomic features are not stable against dose modulation. For a subset of features, it is possible to correct this variability by a simple linear model. However, the correction becomes increasingly less accurate at lower radiation doses.

Our finding that the majority of radiomic features are not stable against varying dose levels is concordant with previously performed phantom studies that demonstrated a marked effect of CT tube current modulation on the value of several radiomic features [7, 8]. Our results are relevant for low-dose lung cancer screening. Globally, low-dose lung cancer screening is a growing trend and our findings underline the importance of standardizing the acquisition process. Ideally, screening and any follow-up examinations should be acquired on the same CT scanner with the same settings. Initiatives to standardize the process are being undertaken [1, 2].

The present results suggest that the most promising feature class regarding robustness is the shape feature class. Previous phantom studies have shown that shape features provide the most promising results regarding robustness against parameter variations (voxel geometry settings, dose level, segmentation of ROI) [20, 21]. We found that first-order features were neither more robust nor more correctable by a linear model than other features. This is in contradiction to Hepp et al and Kim et al who found that first-order features were among the most stable in, respectively, a noise simulation study and a phantom study [10, 17].

From signal-processing theory, we know that a lower radiation dose introduces increasingly random noise to radiomic feature values. This is analogous to how the human visual system perceives lower quality. In other words, increased noise impairs the clinical value of radiomic features. For some features, a lower dose does not lead to noise but to systematic differences that are correctable. The error of a subset of unstable features can very well be explained by a simple linear model (features with a high *R*^2^). This is a promising result for more complicated correction methods. Zhovannik et al used an additive correction model to decrease error in 47 out of 62 feature values with at least a factor of 2 [7]. Wei et al used a 3D generative adversarial network to normalize reduced dose [22]. The decrease in error was significant for 8 out of 9 features. In addition, Mahon et al demonstrated the usage of the ComBat (combatting batch effect) harmonization algorithm, which greatly reduces the variation [20]. It remains to be seen if these methods can function as a uniform correcting method usable in clinical care. The vast number of filters applied to the original image, apart from LoG, does not seem to generate more reproducible features. This raises the question whether there is any need for filters in the already vast amount of radiomic features extracted from the original image. Our finding that features derived from LoG-filtered images are more robust to dose variation is novel and warrants more investigation.

A unique advantage of this study is the radiographic imaging dataset. Fifteen patients underwent a CT scan at four different dose levels sequentially. The nature of the radiographic imaging dataset provides an opportunity to largely isolate variables other than dose levels. To the best of our knowledge, this study is the first multi-dose *in vivo* study on lung nodule radiomic feature reproducibility.

In general, we found shape features to be the most reproducible feature class. Yet, for a feature to be of clinical value, it must improve the diagnostic or prognostic value. Davey et al showed sphericity strongly correlates with overall survival of patients with lung cancer [21]. Yan et al showed that sphericity showed good ability in distinguishing adenocarcinoma from another lung cancer histological type using machine learning [23]. Liu et al found that a model for distinguishing benign from malignant lung nodules based on ten features, among which was the shape feature sphericity, significantly outperformed a clinical variable-based model [23].

Shakir et al found that the shape feature surface volume ratio is most discriminative for nodule classification (benign vs malignant) out of 105 total features, using one-way ANOVA and three supervised selection algorithms [24]. Moreover, they found that the shape feature class had the highest relative contribution in nodule classification out of all the feature classes.

Yang et al selected seven features, among which were the shape features surface volume ratio and elongation, for the best diagnostic performance using hierarchical cluster analysis and the ReliefF method. The value of the conclusions on features with prognostic and/or diagnostic value is limited by slight differences in sets of radiomic features studied compared to this study. Future study needs to confirm if the radiomic features described in the current study have prognostic and/or diagnostic value.

This study has limitations. Manual delineation of the nodules was performed by only one investigator. Previous studies suggested that the standardization by using (semi-) automated segmentation methods provides more robust results [8, 25, 26]. However, the aim of the present study was to investigate if radiomics features are robust against dose modulation. We did not study whether features are sensitive to differences in ROI segmentation. Furthermore, it is known from the literature that this is indeed the case [25, 27, 28]. Therefore, we decided to have only one person segment all the scans. The extent to which segmentation differences interact with radiation dose reduction as to radiomics feature reproducibility is a very interesting question by itself and could very well be a direction for further research. Future studies should preferably be based on multiple delineations by multiple professionals or automation of segmentation. In addition, the high dimensionality of radiomic feature data hinders a simple presentation of results. To complicate the matter, a variety of presentation methods can be found in articles on the topic: ICC, concordance correlation coefficient (CCC), and coefficient of variation (COV) are all used interchangeably. This lack of consistency hinders comparison of results. For this study, we chose to plot bias and *R*^2^ to intuitively visualize trends and calculate the ICC to quantify robustness. Our study counts a relatively small size (32) of nodules studied. This study did not investigate the prognostic or diagnostic value of radiomic features, only the stability of feature values over variations in radiation dose. We recommend further studies to investigate on the stability of radiomic features over different isolated variations such as manual delineation, bin width, or different reconstruction algorithms. The latter might be especially relevant as in a review by Reiazi et al radiation dose was found to be a disruptive parameter in all studies, whereas reconstruction algorithm appeared to be non-disruptive in about 50% of studies [11].

Also, we did not investigate the possible pre-processing of features or scans prior to feature calculation which might further enhance reproducibility [29]. Along the same vein, this study only investigated the reproducibility of radiomic features extracted from FBP constructed scans. Especially at lower milliampere-second levels, iterative reconstruction methods are used to decrease image noise. Shiri et al and Zhao et al showed that the variability and robustness of radiomic features in advanced reconstruction settings are feature-dependent [30, 31].

A solution to the possible lack of robustness of radiomic feature values is to standardize the process of feature extraction and possibly an (inter)national standardization of the clinical radiographic imaging setting. Although the latter seems a bridge too far currently, radiomic feature acquisition standardization initiatives are underway [2]. Finally, although the prespecified nature of radiomics features makes them better explainable/connectable to the underlying biology, we cannot rule out that unsupervised deep learning techniques are less sensitive to variations in radiation dose.

In conclusion, a lower radiation dose introduces increasingly random noise and bias to radiomic feature values of pulmonary nodules. This noise can be corrected for by a linear model for a subset of features. We identified 15% of features as stable according to ICC, with shape as the most robust feature class.

### Supplementary Information

Below is the link to the electronic supplementary material.

Supplementary file1 (PDF 182 KB)

## Abbreviations

ANOVA: Analysis of variance
CCC: Concordance correlation coefficient
COV: Coefficient of variation
FBP: Filtered back projection
GLCM: Gray-level co-occurrence matrix
GLDM: Gray-level dependence matrix
GLRM: Gray-level run length matrix
GLSZM: Gray-level size zone matrix
ICC: Intra-class correlation coefficient
LoG: Laplacian of Gaussian
MSE: Mean square error
MSR: Mean square for rows
ROI: Region of interest

## Declarations

### Guarantor

The scientific guarantor of this publication is Tim Leiner.

### Statistics and biometry

One of the authors has significant statistical expertise.

### Study subjects or cohorts overlap

Some study subjects or cohorts have been previously reported in https://doi.org/10.1097/RCT.0000000000000408.

### Methodology

• Retrospective

• Observational

• Performed at one institution
