# Supplementary material for: Robustness of pulmonary nodule radiomic features on computed tomography as a function of varying radiation dose levels—a multi-dose in vivo patient study
Source: Eur Radiol. 2023 Apr 19;33(10):7044–55. doi: 10.1007/s00330-023-09643-8 (PMC10511375; doi:10.1007/s00330-023-09643-8)
Supplement: Supplementary file 1 — Supplementary file1 (PDF 182 KB) [file 330_2023_9643_MOESM1_ESM.pdf]

Appendix 1:

Inclusion criteria:

- Patients 50 years or older with 1 or more pulmonary nodules scheduled for a follow-up chest CT were eligible for inclusion

Exclusion criteria

- Patients participating in other studies with x-ray exposure were excluded.
- Patients with lung mass (diameter ≥ 3 cm)

Appendix 2:

Filters

1. **Original**: no filter applied
2. **Wavelet**: 8 decompositions per level
3. **LoG**: Laplacian of Gaussian filter, edge enhancement filter. Sigma 2,3,4,5
4. **Square**: squares image intensities, and turns negative values back into negative values after squaring.
5. **SquareRoot**: takes square root of absolute image intensities and turns negative values back into negative values after squaring.
6. **Logarithm**: takes logarithm of (absolute image intensities +1) and turns negative values back into negative values after squaring.
7. **Gradient**: returns the magnitude of the local gradient

Features

1. Shape-based (14 features)
2. First Order Statistics (18 features) * 14 for each filter
3. Gray Level Co-occurrence Matrix (22 features) * 14 for each filter
4. Gray Level Run Length Matrix (16 features) * 14 for each filter
5. Gray Level Size Zone Matrix (16 features) * 14 for each filter
6. Gray Level Dependence Matrix (14 features) * 14 for each filter

Total number of features:

14 + 14 * 86 = 1218

Appendix 3

Table with all ICC values for features extracted from the original image

feature	value
shape_VoxelVolume	0,96
shape_Maximum3DDiameter	0,904
shape_MeshVolume	0,96
shape_MajorAxisLength	0,935
shape_Sphericity	0,807
shape_LeastAxisLength	0,929
shape_Elongation	0,669
shape_SurfaceVolumeRatio	0,839
shape_Maximum2DDiameterSlice	0,928
shape_Flatness	0,708
shape_SurfaceArea	0,939
shape_MinorAxisLength	0,913
shape_Maximum2DDiameterColumn	0,907
shape_Maximum2DDiameterRow	0,92
original_glcm_JointAverage	0,493
original_glcm_JointEntropy	0,726
original_glcm_ClusterShade	0,391
original_glcm_MaximumProbability	0,391
original_glcm_Idmn	0,813
original_glcm_JointEnergy	0,301
original_glcm_Contrast	0,515
original_glcm_DifferenceEntropy	0,422
original_glcm_InverseVariance	0,77
original_glcm_DifferenceVariance	0,362
original_glcm_Idn	0,855
original_glcm_Idm	0,788
original_glcm_Correlation	0,706
original_glcm_Autocorrelation	0,442
original_glcm_SumEntropy	0,58
original_glcm_SumSquares	0,321
original_glcm_ClusterProminence	0,207
original_glcm_Imc2	0,902
original_glcm_Imc1	0,901
original_glcm_DifferenceAverage	0,633
original_glcm_Id	0,774
original_glcm_ClusterTendency	0,304
original_gldm_GrayLevelVariance	0,295
original_gldm_HighGrayLevelEmphasis	0,44
original_gldm_DependenceEntropy	0,836
original_gldm_DependenceNonUniformity	0,74
original_gldm_GrayLevelNonUniformity	0,952
original_gldm_SmallDependenceEmphasis	0,768

original_gldm_SmallDependenceHighGrayLevelEmphasis	0,317
original_gldm_DependenceNonUniformityNormalized	0,83
original_gldm_LargeDependenceEmphasis	0,763
original_gldm_LargeDependenceLowGrayLevelEmphasis	0,353
original_gldm_DependenceVariance	0,729
original_gldm_LargeDependenceHighGrayLevelEmphasis	0,686
original_gldm_SmallDependenceLowGrayLevelEmphasis	0,651
original_gldm_LowGrayLevelEmphasis	0,723
original_firstorder_InterquartileRange	0,43
original_firstorder_Skewness	0,692
original_firstorder_Uniformity	0,603
original_firstorder_Median	0,856
original_firstorder_Energy	0,697
original_firstorder_RobustMeanAbsoluteDeviation	0,425
original_firstorder_MeanAbsoluteDeviation	0,355
original_firstorder_TotalEnergy	0,68
original_firstorder_Maximum	0,761
original_firstorder_RootMeanSquared	0,752
original_firstorder_90Percentile	0,881
original_firstorder_Minimum	0,321
original_firstorder_Entropy	0,541
original_firstorder_Range	0,394
original_firstorder_Variance	0,293
original_firstorder_10Percentile	0,608
original_firstorder_Kurtosis	0,608
original_firstorder_Mean	0,83
original_glrlm_ShortRunLowGrayLevelEmphasis	0,727
original_glrlm_GrayLevelVariance	0,285
original_glrlm_LowGrayLevelRunEmphasis	0,723
original_glrlm_GrayLevelNonUniformityNormalized	0,597
original_glrlm_RunVariance	0,789
original_glrlm_GrayLevelNonUniformity	0,966
original_glrlm_LongRunEmphasis	0,807
original_glrlm_ShortRunHighGrayLevelEmphasis	0,413
original_glrlm_RunLengthNonUniformity	0,885
original_glrlm_ShortRunEmphasis	0,843
original_glrlm_LongRunHighGrayLevelEmphasis	0,53
original_glrlm_RunPercentage	0,845
original_glrlm_LongRunLowGrayLevelEmphasis	0,701
original_glrlm_RunEntropy	0,596
original_glrlm_HighGrayLevelRunEmphasis	0,435
original_glrlm_RunLengthNonUniformityNormalized	0,854
original_glszm_GrayLevelVariance	0,25
original_glszm_ZoneVariance	0,653
original_glszm_GrayLevelNonUniformityNormalized	0,476
original_glszm_SizeZoneNonUniformityNormalized	0,321

original_glszm_SizeZoneNonUniformity	0,635
original_glszm_GrayLevelNonUniformity	0,902
original_glszm_LargeAreaEmphasis	0,662
original_glszm_SmallAreaHighGrayLevelEmphasis	0,323
original_glszm_ZonePercentage	0,845
original_glszm_LargeAreaLowGrayLevelEmphasis	0,425
original_glszm_LargeAreaHighGrayLevelEmphasis	0,63
original_glszm_HighGrayLevelZoneEmphasis	0,363
original_glszm_SmallAreaEmphasis	0,27
original_glszm_LowGrayLevelZoneEmphasis	0,638
original_glszm_ZoneEntropy	0,769
original_glszm_SmallAreaLowGrayLevelEmphasis	0,5

Appendix 4

Table with negative slope features

feature	slope
square_glszm_SizeZoneNonUniformityNormalized	-0,052
square_glszm_SizeZoneNonUniformityNormalized	-0,157
square_glszm_SizeZoneNonUniformity	-0,007
square_glszm_SmallAreaEmphasis	-0,07
square_glszm_LowGrayLevelZoneEmphasis	-0,045
square_glszm_LowGrayLevelZoneEmphasis	-0,018
log-sigma-3-0-mm-3D_glszm_SmallAreaLowGrayLevelEmphasis	-0,004
log-sigma-3-0-mm-3D_glszm_SmallAreaLowGrayLevelEmphasis	-0,2
log-sigma-4-0-mm-3D_glszm_SmallAreaLowGrayLevelEmphasis	-0,022
wavelet-LHH_glcm_ClusterShade	-0,05
wavelet-LHH_glcm_ClusterShade	-0,082
wavelet-LHH_glcm_ClusterShade	-0,966
wavelet-LHH_firstorder_Skewness	-0,003
wavelet-LHH_firstorder_Skewness	-0,079
wavelet-LHH_firstorder_Median	-0,042
wavelet-HLH_glcm_ClusterShade	-0,201
wavelet-HLH_glcm_JointEnergy	-0,005
wavelet-HLH_firstorder_Skewness	-0,029
wavelet-HLH_firstorder_Skewness	-0,301
wavelet-HLH_firstorder_Skewness	-0,106
wavelet-HLH_glszm_SmallAreaLowGrayLevelEmphasis	-0,017
wavelet-HHH_glcm_ClusterShade	-0,108
wavelet-HHH_glcm_Idmn	-0,016
wavelet-HHH_glcm_InverseVariance	-0,079
wavelet-HHH_glcm_InverseVariance	-0,07
wavelet-HHH_firstorder_Skewness	-0,297
wavelet-HHH_firstorder_Skewness	-0,123
wavelet-HHH_firstorder_Median	-0,013
wavelet-HHH_firstorder_Median	-0,017
wavelet-HHH_firstorder_Kurtosis	-0,132
wavelet-HHH_firstorder_Mean	-0,033
wavelet-HHH_glszm_SmallAreaLowGrayLevelEmphasis	-0,022
wavelet-HHL_glcm_ClusterShade	-0,193
wavelet-HHL_glcm_ClusterShade	-0,035
wavelet-HHL_glcm_Correlation	-0,094
wavelet-HHL_firstorder_Skewness	-0,238
wavelet-HHL_firstorder_Skewness	-0,034
wavelet-HHL_firstorder_Skewness	-0,038
wavelet-HHL_firstorder_Median	-0,135
wavelet-HHL_firstorder_Median	-0,098
wavelet-HHL_glszm_SizeZoneNonUniformityNormalized	-0,069
wavelet-HHL_glszm_SmallAreaEmphasis	-0,374
